# Impact of Health Education Interventions for Control of *Taenia solium* Cysticercosis/Taeniasis in Endemic Countries: A Systematic Review

**DOI:** 10.1002/puh2.70147

**Published:** 2025-10-24

**Authors:** Chacha Nyangi, Ernatus Martin Mkupasi, Helena Aminiel Ngowi, Christopher Mahonge, Andrea Sylvia Winkler

**Affiliations:** ^1^ Department of Veterinary Medicine and Public Health Sokoine University of Agriculture Morogoro Tanzania; ^2^ Department of Food Science and Technology Mbeya University of Science and Technology Mbeya Tanzania; ^3^ Department of Policy, Planning and Management Sokoine University of Agriculture Morogoro Tanzania; ^4^ Department of Neurology, TUM University Hospital, and Center for Global Health School of Medicine and Health Technical University of Munich (TUM), Germany; ^5^ Department of Community Medicine and Global Health, Institute of Health and Society University of Oslo Norway

**Keywords:** behavioural change, cysticercosis, disease prevalence, health education, knowledge, systematic review, *taenia solium*

## Abstract

Despite some control efforts, *Taenia solium* cysticercosis/taeniasis (TSCT) remains widespread in many low‐income countries across sub‐Saharan Africa, Latin America and Asia. With increased global interaction, the risk of infection also rises in high‐income countries (HICs) and middle‐income countries (MICs). Community knowledge and awareness are crucial to influence behavioural change and thus aid in controlling the parasite. This systematic review examined the effectiveness of health education interventions in managing TSCT to inform future disease control strategies. Papers published up to June 2024 were searched through PubMed and Google search engines. Studies evaluating interventions involving health education aimed at improving knowledge, attitudes and practices (KAPs) to alter behavioural responses regarding TSCT were included. Initially, 392 studies were identified, with 21 publications ultimately included in this review. Although behavioural changes and reductions in disease prevalence were challenging to evaluate across the 21 studies, most concluded that health education, developed with community participation, enhanced KAPs, modified behaviour and reduced disease prevalence in the short term.

## Graphical abstract

This systematic review assessed health education interventions for controlling *Taenia solium* taeniasis/cysticercosis. Of 392 studies screened, 21 met the inclusion criteria. Findings showed that community‐based education improved knowledge, attitudes and practices, with short‐term behavioural changes and reduced prevalence, although long‐term impact remained difficult to evaluate.

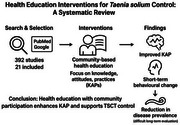

## 1 Introduction

*Taenia solium* cysticercosis/taeniasis (TSCT) is a neglected zoonotic disease that poses substantial economic and public health challenges, particularly in low‐ and middle‐income countries (LMICs) across Asia, Latin America and sub‐Saharan Africa [1]. TSCT affects 50 million people worldwide [2]. It occurs more frequently in countries with low socio‐economic status, particularly in areas with poor personal hygiene, inadequate environmental sanitation, poor pig management, inadequate meat inspection and a lack of knowledge about TSCT [3, 4, 5, 6]. According to the FAO and WHO (2014), *T. solium* is the foodborne parasite of greatest concern worldwide. According to reports, *T. solium* neurocysticercosis was the cause of more than 10% of acute case admissions to neurological departments in endemic countries [7]. In these regions, it is among the leading preventable causes of neurological disorders. Due to globalisation with increased mobility of humans and goods, the parasites have even been reported in high‐income countries (HICs) [8, 9]. The movement of people from regions where the *T. solium* tapeworm is common to areas where it is not is the main reason for the recent appearance of cysticercosis in developed nations like the United States and Europe [10]. Migrants can unwittingly carry the parasite and introduce it to new populations, creating a serious public health risk [11]. The transmission of the disease through immigration is a complicated issue, influenced by a combination of social, economic, cultural and public health dynamics.

Recommended control strategies have failed to eliminate TSCT in the current endemic countries. Proper maintenance and total use of latrines have been far from being achieved in many low‐income areas, especially in rural settings. Cultural and taboos obstacles regarding the construction and use of toilets need to be addressed through community‐based and participatory approaches [12, 13, 14]. Treatment of taeniasis cases with praziquantel or niclosamide is not regularly practised due to a lack of diagnostic capacities, and TSCT is not being considered among differential diagnoses by clinicians because of a lack of training. Moreover, due to low socio‐economic development and awareness about the problem, many people do not visit healthcare facilities [15, 16]. Personal hygiene practices, such as proper handwashing after defecation and before eating, can prevent the acquisition of TSCT and many other diseases; however, these practices are not followed due to cultural norms or the limited availability of clean and safe water in most rural areas [17, 18]. Community health education should be considered for sustainable control of the parasite, and school children should be a primary target [17, 19, 20]. Although oxfendazole is successful in treating infected pigs, the medication is not easily accessible in these nations, and many vets are unaware of its existence. Additionally, it is costly and difficult to diagnose TSCT in live pigs [21]. Improvement in pig husbandry and appropriate meat inspection and control was crucial in eliminating the infection in HICs but has limited effect in current endemic countries due to traditional pig rearing, lack of centralised slaughter and inadequate qualified meat inspectors [17].

Lack of knowledge is reported to be the main barrier to the effective control of TSCT [22]. Increased community knowledge regarding the TSCT is presumed necessary to enhance adherence to disease control measures and change of risky behaviours, resulting in an effective and sustainable control of TSCT [5, 14, 19, 20, 22, 23, 24]. Health education, if properly designed and implemented, can be effective in knowledge delivery, resulting in compliance with disease control measures of TSCT and many other infectious diseases [19]. Health education can focus on various aspects such as the biology of the disease, transmission and control options [1].

Information regarding impacts and challenges of the applied health education interventions in control of TSCT is important to inform policymakers and guide development and implementation of new interventions. However, no study has assessed the impact of applied health education interventions in the control of TSCT for better planning and elimination of the parasite. This work summarises the effects of health education interventions in controlling TSCT in terms of change in knowledge, attitude, practices (KAPs), behaviour and disease burden.

## 2 Methodology

### 2.1 Information Search and Selection Criteria

English‐language publications on health education studies for the control of TSCT, as of June 2024, were searched using three search engines: PubMed, Google Scholar and others. As not all relevant publications were retrieved from the three search engines mentioned earlier, we also conducted direct searches in the following journals: African Journal Online, BMC Public Health, Taylor & Francis Online and the American Journal of Health Education. The following search terms were used: *impact, effectiveness*, *health education, cysticercosis, taeniasis/taeniasis and T. solium*.

### 2.2 Systematic Review Process

The systematic review was carried out according to a pre‐registered protocol with the International Prospective Register of Systematic Reviews (PROSPERO CRD 42021291640) and reported following the Preferred Reporting Items for Systematic Reviews and Meta‐Analyses (PRISMA) guidelines [25]. Two reviewers (C.N. and H.A.N.) performed the selection process independently. After screening abstracts and titles, the same reviewers retrieved full‐text reports for a comprehensive assessment against the inclusion criteria. A title or abstract of an article containing the key search terms was likely to be eligible for inclusion. In those cases, the full article was obtained and assessed for relevance. Criteria for assessing the methodological quality of RCT studies included withdrawal/dropout rates, study design, randomisation, inclusion of a control group and sample size [26].

### 2.3 Inclusion/Exclusion Criteria

The inclusion criteria included both published and unpublished studies that assessed the effectiveness/impact of the health education intervention on KAP, behavioural change and control of TSCT. The exclusion criteria included duplicates and studies that combined health education with other interventions, making it challenging to isolate the specific effect of the health education intervention. A meta‐analysis was not conducted due to the absence of quantitative results in some studies and the lack of statistical significance indicators in several trials.

## 3 Results

**FIGURE 1:**
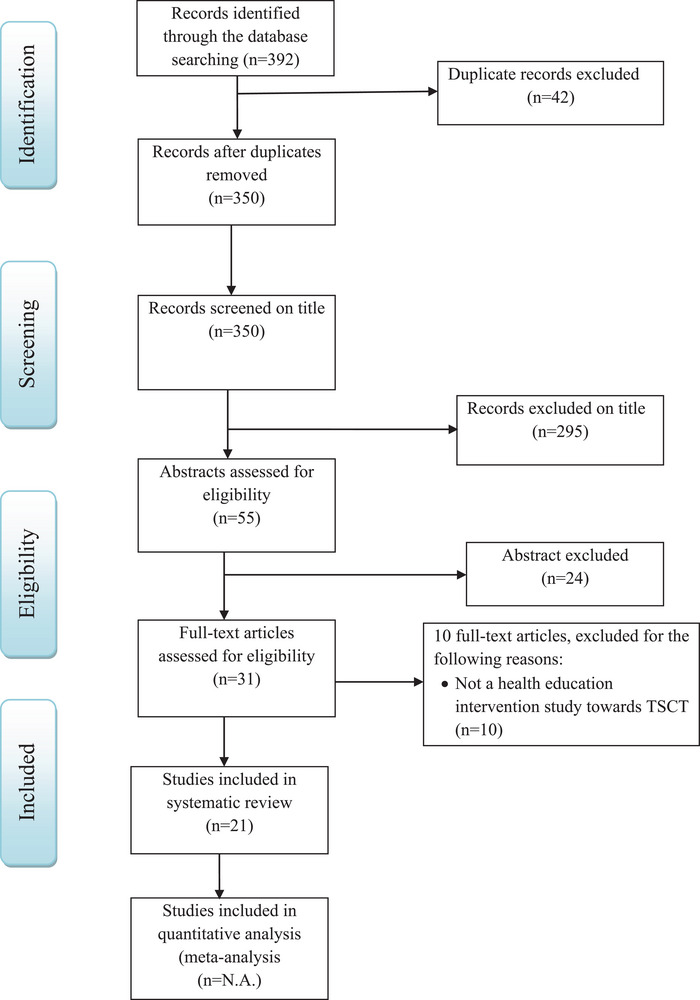
PRISMA diagram for a systematic review of impact of health education interventions in control of *Taenia solium* cysticercosis/taeniasis in endemic countries as of June 2024.

**FIGURE 2:**
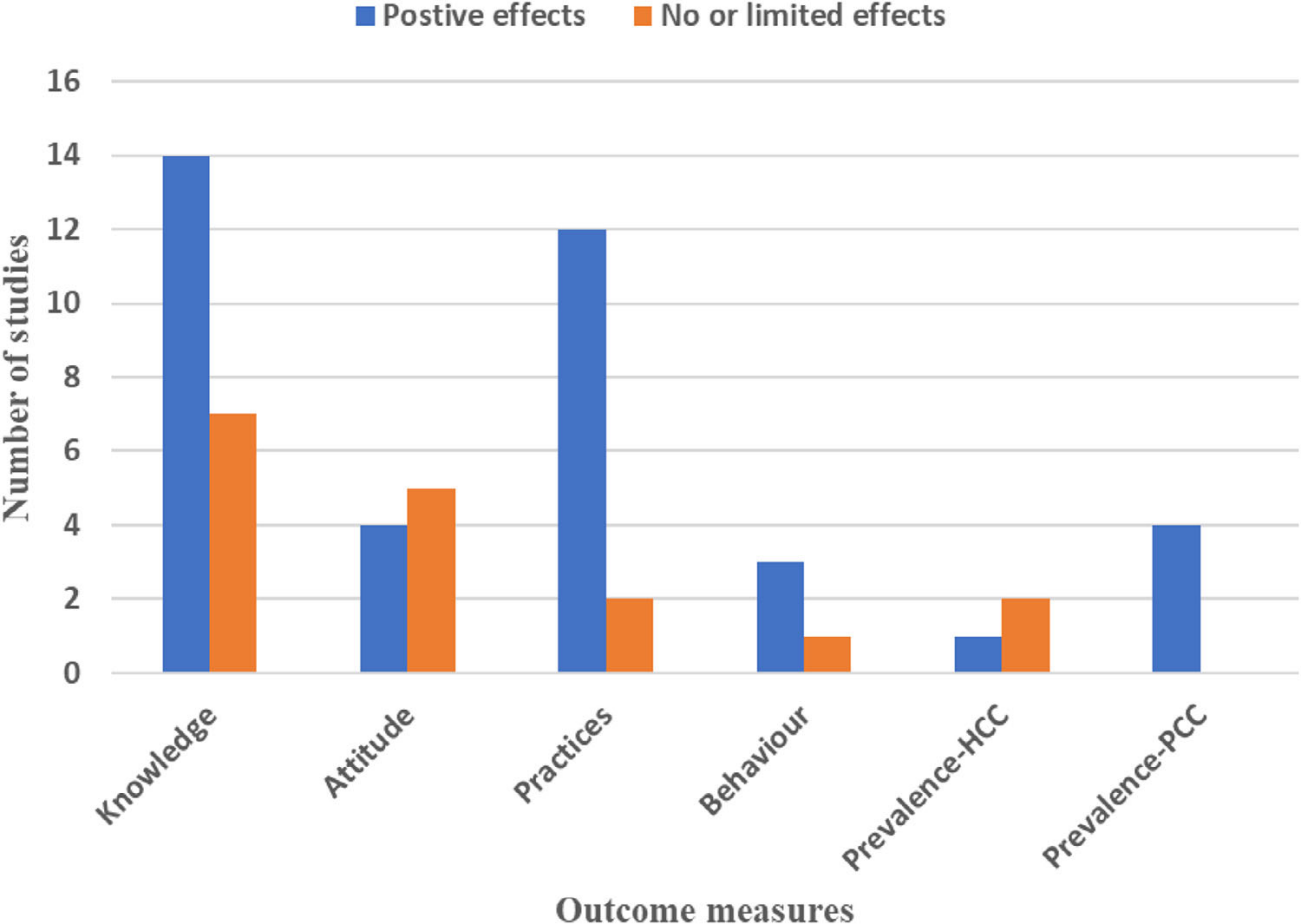
The impact of health education intervention as revealed during a review of literature from studies (*n* = 21) as of June 2024 based on Section 2.3).

A total of 392 papers were retrieved, and 371 were excluded on the basis of the reasons outlined in Figure 1. The review and selection process resulted in 21 articles that fulfilled the inclusion criteria (Table 1). All of these papers focused on the impact of health education interventions in controlling TSCT. The intended outcomes of the interventions are summarised in terms of KAPs, behaviour and change in TSCT burden (Table 1 and Figure 2).

**TABLE 1:** A summary of the aims, population/coverage and major outcomes of the 21 studies included in this review.

Authors	Country	Follow‐up period	Study aims	Population and sample size	Study design	Outcome measures
Ertel et al. [27]	Tanzania	2 weeks	Assessment of the effect of ‘The Vicious Worm’ on knowledge uptake among professionals and to investigate their attitudes towards the programme	Students and professionals (*n* = 79)	Pre‐ and post‐test design, no control (using vicious worms, a computer‐based health educational programme)	**Knowledge**: Significantly improved (*p* < 0.001) **Attitude**: Not assessed **Practices**: Not assessed **Behaviour**: Not assessed **PCC**: Not assessed
Ngowi et al. [5]	Tanzania	10–12 months	Estimate the effectiveness of the health‐education intervention in reducing the incidence rate of PCC and improving knowledge and practices related to the transmission of PCC	836 pig farmers	RCT (CONSORT guideline)	**Knowledge**: Improvement in control and intervention group >42% **Attitude**: Not assessed **Practices**: No significant improvement **PCC**: Reduction in incidence rate of PCC **Behaviour**: • Significant reduction in consumption of infective pork in intervention group (by 20%, *p* = 0.005), whereas there was a significant increase in consumption of infective pork in the control group • Household selling pigs infected with cysticercosis increased significantly in the intervention group • Significant increase in the consumption of infected pork by the control group
Ngowi et al. [28]	Tanzania	1 year	Application of a health promotion model to implement and evaluate evidence‐based strategies for control of *Taenia solium* infections	52% (434) of pig farmers at baseline and 48% (393) of pig farmers in the follow‐up study	RCT (PRECEDE–PROCEED model)	**Knowledge**: No significant improvement **Attitude**: Not assessed **Practices**: Not assessed **PCC**: 43% reduction in the incidence rate of PCC in the intervention group **Behaviour**: Significant reduction in consumption of infective pork (20%) in intervention group (*p* = 0.005), whereas there was a significant increase in consumption of infective pork in the control group
Ngowi et al. [29]	Tanzania	5 months	Assessment of the effect of health education intervention on knowledge and attitudes related to *T. solium* transmission and control	Smallholder pig farmers in Iringa Rural (*n* = 750) and Chunya (*n* = 700) districts	Quasi‐experimental study design with pre‐ and post‐intervention assessments of the same respondents to obtain paired data	**Knowledge**: Significant improvements in knowledge related to transmission and control of *T. solium* (*p* < 0.001) **Attitude**: Significant improvements in attitude related to transmission and control of *T. solium* (*p* < 0.001) **Practices**: Not assessed **PCC**: Not assessed **Behaviour**: Not assessed
Mwidunda et al. [20]	Tanzania	1 year	Assessment of knowledge and attitudes related to *T. solium* cysticercosis and taeniasis in Primary and Secondary schools from an endemic area	Total of 2350 students (1126 primary school and 1224 secondary school)	Cluster RCT following CONSORT guideline (with both pre‐ and post‐intervention assessments)	**Knowledge**: Improvement in knowledge regarding taeniasis, PCC, human cysticercosis and epilepsy **Attitude**: • Improvement in the condemnation of infected pigs • Reduce the attitude of contacting a veterinarian if the pig is found to be infected **Practices**: Not assessed **Behaviour**: Not assessed
Wohlgemut et al. [30]	Kenya	2 years	Determine whether farmer training workshops followed by one‐on‐one training were associated with the acquisition of knowledge and cooking methods for pork that are effective in reducing transmission	282 pig farmers	Not indicated. No randomisation	**Knowledge**: Increased for those who attended the workshop (*p* = 0.005) **Attitude**: Not assessed **Practices**: Significant increase in tethering (*p* < 0.001) **Behaviour**: Not assessed
Hobbs et al. [31]	Zambia	1 day	Evaluate the effects of ‘The Vicious Worm’ on *T. solium‐*associated knowledge uptake in primary school students from the highly endemic Province of Zambia	99 students	Pre‐ and post‐test design, no control	**Knowledge**: Significantly improved (*p* = 0.005) **Attitude**: Not assessed **Practices**: Not assessed **Behaviour**: Not assessed
Carabin et al. [23]	Burkina Faso	3 years	Estimate the effectiveness of a community‐based educational intervention in reducing the frequency of human cysticercosis	3566 participants at baseline and 1035 participants at all visits	Cluster—RCT following the CONSORT guideline and the PRECEDE–PROCEED model	**Knowledge**: Not assessed **Attitude**: Not assessed **Practices**: An increase in latrine construction in the intervention villages **Behaviour**: Not assessed **Prevalence of HCC**: Decrease in prevalence of active HCC
Sarti et al. [24]	Mexico	6 months	Evaluate health education as a strategy for the prevention of taeniasis and cysticercosis	146 participants	Pre‐ and post‐test design, randomisation and no control	**Knowledge**: Significantly increased by 25%–30% **Attitude**: Not assessed **Practices**: Significant reduction in free‐range pigs by 50% **Behaviour**: Improved **Prevalence**: • **PCC**: From 2.6% (lingual examination) and 5.2% (immunoblot assay) to 0% and 1.2%, respectively (*p* < 0.05) 6 months later • **Human taeniasis**: No significant change
Alexander et al. [19]	India	6 months	Evaluate the effectiveness of a comprehensive health education programme on the knowledge and practices of high‐school children and the general community regarding taeniasis and cysticercosis	1060 participants (school children, farmers and community members)	Pre‐ and post‐test design, randomisation and no control	**Knowledge**: Increased by 46% (*p* < 0.0001) **Awareness**: Improved x3 **Attitude**: Not assessed **Practices**: Hand washing before eating improved (x4.8) and after visiting the latrine (x3.6) (*p* < 0.0001) **Behaviour**: • Pork consumption decreased by 45% (*p* < 0.001). • Open field defecation decreased by 23% (*p* < 0.001)
Veena et al. [32]	India	3 months	To generate material; and to assess the feasibility, and efficacy of such a project in India in increasing awareness about taeniasis and neurocysticercosis	200 teachers, 4786 students, 846 parents, 79 sanitary staff, 65 school‐health nurses and 23 school‐health physicians	Pre‐ and post‐test design, randomisation and no control	**Knowledge**: Significant improvement in teachers’ knowledge about the impact of changing sanitary habits in the prevention of the disease **Attitude**: Not assessed **Practices**: Not assessed
Vaernewyck et al. [33]	Zambia	3 weeks	To assess the effects of health education using ‘The Vicious Worm’ among Zambian pork supply chain workers	Pork supply chain workers at the level of live trading, slaughtering, meat cutting and distribution	Pre‐ and post‐test design, no control	**Knowledge**: About *T*. *solium* was significantly higher (*p* < 0.001) both immediately after, and 3 weeks later
Beam et al. [34]	Peru	4 months	To educate community members on *T. solium* transmission and motivate participation in community‐led prevention and control	226 people at baseline interview and 191 at follow‐up interview	Pre‐ and post‐test design, no control	**Knowledge**: Knowledge of the life cycle increased significantly after the workshop, with greater gains for workshop attendees than non‐attendees
Chilundo et al. [35]	Mozambique	24 months	To evaluate the effectiveness of pig farming education	100 smallholders pig farmers at baseline and 90 at follow up	Community‐based animal health education intervention trial	**Knowledge**: Significant improvement regarding pig pen design (*p* = 0.014), reasons for confine the pigs (*p* = 0.016), as well as the adoption of the new introduced pig pen model (*p* = 0.025) **Practices**: Significant improvement in practices of acceptable, good hygiene of the pig pen (*p* = 0.009 and *p* = 0.014, respectively), in both between trained and non‐trained groups
Nyangi et al. [36]	Tanzania	1 day	To assess the effectiveness of the health education intervention towards improving community knowledge, attitude and practices (KAP) in controlling TSCT	78 pig farmers, non‐pig farmers and ToTs during pre‐intervention and post‐intervention	Pre‐ and post‐intervention study design	**Knowledge**: Non‐significant improvement in knowledge and significant improvement in knowledge regarding a link between PCC and epilepsy (*p* < 0.001) **Practices**: Significant improvement in practices of washing vegetables and fruits (*p* = 0.025)
Makingi et al. [37]	Tanzania	12 months	Community Health‐Education Intervention Trial against Human *Taenia solium* Taeniasis/Cysticercosis in Central and Southern Zones of Tanzania	42 villages (21 intervention group and 21 control group) and 872 participants	Cluster‐randomised control trial with pre‐ and post‐intervention	**Knowledge**: • No significant difference in mean knowledge scores between the control and intervention groups at baseline • There were significantly higher knowledge mean scores in the intervention group compared to the control group at 1‐year post‐intervention (*p* < 0.001). **Practices**: • No significant difference in mean practice scores between the control and intervention groups at baseline • No significant difference in the mean practice scores between the intervention and the control group at 1‐year post‐intervention (*p* = 0.31). **Prevalence**: • **HCC**: No significant difference between the intervention and the control group after 1 year. • **Taeniasis**: No significant difference between the intervention and the control group at the baseline (*p* = 0.97)
Wilson et al. [38]	Tanzania	12 months	To assess the effectiveness of the health education intervention in reducing the prevalence of PCC and enhancing pig farmers’ KAP related to the control of PCC in Kongwa and Songwe Districts in Tanzania	Total of 692 and 486 respondents interviewed and a total 692 and 317 pigs were sampled at baseline and post‐intervention, respectively	Cluster‐randomised control trial with pre‐ and post‐intervention	**Knowledge**: Significant increase in knowledge of transmission (*p* < 0.001) in the treatment group **Attitude**: Significant increase in attitude level towards risk of free‐range pig (*p* < 0.001), increase the ability to identify measles pork (*p* < 0.001) and an increase in the willingness to condemn infected pork (*p* < 0.001) in the treatment group **Practices**: Significant reduction in open defecation (*p* < 0.001) in the intervention group and significant increase in indoor pig management (*p* = 0.004) in the control group **PCC prevalence**: Reduction of PCC in the experimental group compared to the control group although the reduction effect was not statistically significant (*p* = 0.518)
Kajuna et al. [39]	Tanzania	25 months	To assess the effects of developed digital health literacy content on PCC prevalence, pig‐keeping style and pig pen and latrine qualities	346 pigs were examined at baseline, and 298 pigs during post‐intervention	A quasi‐controlled field trial with pre‐ and post‐intervention	**Knowledge**: Not assessed **Attitude**: Not assessed **Practices**: • Significantly increased in pig confinement (*p* = 0.026) and pig pen quality (*p* = 0.025) • However, the quality of household latrines (*p* = 0.453) was not improved **PCC prevalence**: No significant effect on the prevalence of PCC (*p* = 0.231)
Holst et al. [40]	Tanzania	12 months	To assess the effect of a digital health education intervention on the uptake and retention of knowledge related to HIV/AIDS, tuberculosis (TB) and TSCT in rural communities in Iringa, Tanzania	A total of 600 participants were recruited into the intervention group (*n* = 298) and the control group (*n* = 302)	Non‐randomised intervention study	**Knowledge**: • At baseline, no statistically significant difference in knowledge of *T. solium* was observed • At 12 months after intervention, knowledge about *T. solium* (neuro) cysticercosis and taeniasis was higher in the intervention group than in the control group
Chen et al. [41]	China	48 months	To investigate the changes in the awareness rate of TSCT control knowledge among medical professionals	A total of 663 medical professionals were investigated in 474 in the intervention and 189 in the control group	Pre‐ and post‐intervention study design	**Knowledge**: • Results from 2016 reported no significant differences in awareness between the intervention and control groups • Results from the 2020 study showed a higher awareness rate of TSCT control knowledge in the intervention group than in the control group
Deng et al. [42]	China	36 months	To compare the changes in KAP related to taeniasis and cysticercosis among primary school students		Pre‐ and post‐intervention study design	**Knowledge**: • Significant difference in the awareness of taeniasis and cysticercosis control knowledge between male and female students (*p* = 0.01) • Significant increase in knowledge regarding cysticercosis control (*p* < 0.001) **Attitude**: Significant decrease in the proportion of students with a habit of eating raw or undercooked meat (*p* < 0.001) **Practices**: Improved in handwashing before meals and after using toilet

Abbreviations: CONSORT, Consolidated Standards of Reporting Trials; HCC, human cysticercosis; PCC, porcine cysticercosis; PRECEDE–PROCEED model, Predisposing, Reinforcing and Enabling Constructs in Educational Diagnosis and Evaluation‐Policy, Regulatory and Organisational Constructs in Educational and Environmental Development; RCT, Randomised Control Trial; TSCT, *Taenia solium* cysticercosis/taeniasis.

### 3.1 Effects of Health Education on Knowledge and Attitudes

Fifteen out of the 21 studies reported that health education interventions increased study population's knowledge significantly [5, 19, 20, 24, 27, 29, 30, 31, 32, 33, 34, 35, 38, 40, 42] (Table 1).

Four studies evaluated attitudes [20, 29, 38, 42]. Ngowi et al. [29] reported a significant improvement in attitude related to transmission and control of *T. solium* (*p* < 0.001); Mwidunda et al. [20] reported that a positive attitude towards the condemnation of infected meat was observed, but there was a negative attitude towards seeking veterinary assistance if a pig was found to be infected with cysticercosis in a school‐based cluster randomised health education intervention. Wilson et al. [38] reported a significant increase in positive attitude towards the risk of free‐range pig (*p* < 0.001) and in the willingness to condemn infected pork (*p* < 0.001) in the treatment group. Deng et al. [42] reported a significant decrease in the proportion of students with a habit of eating raw or undercooked meat (*p* < 0.001) (Table 1).

### 3.2 Effects of Health Education on Practices Related to TSCT

Eleven studies evaluated changes in practices related to TSCT transmission, open defecation, pig confinement, washing fruits and vegetables, eating raw or undercooked pork and handwashing before meals and after toilet use. Three studies reported statistically significant behaviour change [5, 19, 28], whereas eight studies observed non‐statistically significant behaviour change [23, 24, 30, 32, 33, 36, 38, 39, 42].

### 3.3 Effects of Health Education on Human and Porcine Cysticercosis Burdens

A study by Carabin et al. [23] reported a statistically significant decrease in the prevalence of active human cysticercosis (HCC), whereas a study by Ngowi et al. [5] reported a reduction in the incidence rate of porcine cysticercosis. Additionally, although not a randomised trial, a study by Sarti et al. [24] reported a statistically significant decrease in porcine cysticercosis with no significant change in human taeniasis prevalence (Table 1).

### 3.4 Quality of the Studies

Out of 21 studies reviewed, only six of the studies incorporated an RCT [5, 20, 23, 28, 37, 38]. Seven studies did not incorporate or report a control arm [19, 24, 27, 31, 32, 33, 34], and one study with a control arm did not allocate it randomly [30] (Table 1). Most of the studies did not abide by Consolidated Standards of Reporting Trials (CONSORT) reporting standards [43]. Additionally, we ranked the RCT studies (Table 2) according to criteria defined by Jadad et al. [26]. The non‐RCT studies were reported as controlled pre‐ and post‐test designs or quasi‐experimental designs. In a quasi‐experimental design, the goal is to establish a cause‐and‐effect relationship between an independent and dependent variable. Unlike a true experiment, a quasi‐experiment design lacks randomisation, so they can be considered as RCTs if they are randomised; instead, subjects are assigned to groups based on specific, non‐random criteria [44]. The results from the six RCT studies were of reasonable validity [5, 20, 23, 28, 37, 38] (Table 2), whereas those from the non‐RCT studies have to be considered carefully, as the validity and the generalisability of these trials are limited [19, 24, 27, 29, 30, 31]. Due to reduced statistical power, the absence of a control group or poor study design [44], almost all non‐RCT studies employed a pre‐ and post‐test design without a control. Additionally, three studies [31, 33, 34] used pre‐ and post‐test designs without randomisation or a control group. Other identified weaknesses in the study design and implementation included small sample sizes in six studies, which may have compromised the statistical power [24, 27, 30, 33, 34, 35].

**TABLE 2:** Method quality of the reviewed Randomised Control Trial (RCT) studies ranked according to criteria defined by Jadad et al. [26].

Rank	Study	Randomisation	Randomisation point	Withdrawals/Dropouts	Withdrawals/Dropouts points	Total points^a^	Limitations
1	Ngowi et al. [5]	Yes, two‐stage sampling Procedure	2	Yes, reasons reported	1	3	• Difficulty in controlling the experimental conditions • Short follow‐up period of pigs (median 4 months) • Small sample sizes in many villages studied
2	Ngowi et al. [28]	Yes, two stage sampling Procedure	2	Yes, reasons reported	1	3	• Lack of interventions at a broader environmental level such as influence of public policies • Lack of adequate resources limit broader implementation of the project • *p* values not reported
3	Mwidunda et al. [20]	Yes, two stage sampling Procedure	2	Yes, reasons reported	1	3	• Participants dropout and exclusion of five schools • The relatively few numbers of items used in measuring knowledge and attitude might have posed limitations to the inferences drawn • *p* values not reported
4	Carabin et al. [23]	Yes, two stage sampling Procedure	2	Yes, reasons reported	1	3	• The baseline prevalence of active cysticercosis was not balanced between the control and intervention villages • Large number of individuals lost to follow‐up during the 3 years of the study because of the discovery of gold in the area (*p* > 0.05)
5	Makingi et al. [37]	Yes, two stage sampling Procedure	2	Yes, reasons reported	1	3	• Two hundred and ten households lost to follow‐up due to various reasons, including death and migration to other areas, and refusal of some of the respondents to continue
6	Wilson et al. [38]	Yes, two stage sampling Procedure	2	Yes, reasons reported	1	3	• Loss of pigs reported to having succumb due to African swine fever • Lack of practical section on pig feeding and housing structure

^a^ Without double‐blinding criteria; maximum ranking possible = 3 points.

## 4 Discussion

The reviewed studies indicate that health education is a cornerstone of the TSTC control. The studies varied in design, participants and observation period, making comparison difficult. However, most concluded that health education interventions are promising with positive impacts on the control of TSCT. The results of most studies reviewed show that health education interventions improved KAPs related to TSCT and reduced the burden of the disease, as exemplified by the prevalence of human and porcine cysticercosis. However, the long‐term impact of the community health education has not been evaluated.

It is worth noting that most of the reviewed studies concluded that targeted communities well received health education interventions and that it is a promising and effective strategy for *T. solium* control. Health education interventions were varied and include pre‐ and post‐test design, with no control (using ‘The Vicious Worms’ a computer‐based health educational programme), RCT (CONSORT guideline), RCT (PRECEDE–PROCEED model), quasi‐experimental study design with pre‐ and post‐intervention assessments of the same respondents to obtain paired data, cluster RCT following CONSORT guideline (with both pre‐ and post‐intervention assessments), pre‐ and post‐test design with no control, cluster—RCT following CONSORT guideline and PRECEDE–PROCEED model, pre‐ and post‐test design, randomisation and no control, and community‐based animal health education intervention trial.

The findings of this review align with several studies indicating the potential of health education to improve KAPs regarding TSCT. For example, several studies in Tanzania observed an improvement in knowledge about at least one of the aspects of TSCT. Ngowi et al. [5] reported a 42% improvement in knowledge about *T. solium* following health education, although behavioural changes were more difficult to assess. Similarly, Mwidunda et al. [20] found significant improvements in students’ knowledge and attitudes towards *T. solium*, but behavioural changes were less pronounced. Ertel et al. [27], Ngowi et al. [29], Wilson et al. [38] and Holst et al. [40] all conducted their study and reported an improvement in knowledge about TSCT. Several other studies across the globe observed the same trend [19, 24, 30, 31, 32, 33, 34, 35, 36, 38, 40, 41, 42]. This reflects the present review, where knowledge improvements were consistently reported, but long‐term behaviour changes were harder to evaluate.

Three studies looked directly at behaviour change and found that health education interventions may influence it. The first study showed a significant decrease in the consumption of infected pork in the intervention group, whereas the control group experienced a notable increase in consumption [5]. Additionally, there was a substantial rise in the number of households selling pigs infected with cysticercosis [5]. A second study reported a 20% reduction in the consumption of infected pork in the intervention group (*p* = 0.005). At the same time, there was a considerable increase in the consumption of infective pork in the control group [28]. A third study reported a significant decrease in pork consumption and open field defecation [19].

A recurring theme from the reviewed studies is the challenge of measuring behaviour change and reductions in disease prevalence. Carabin et al. [23] noted that although some interventions led to significant knowledge gains, quantifying changes in practices such as handwashing and latrine use was challenging. However, Sarti et al. [24] found that their health education programme significantly reduced free‐range pig farming, a critical risk factor for *T. solium* transmission. This underscores the importance of tailoring health education strategies to address specific local behaviours that need modification.

Additionally, community‐based approaches, as evidenced by Sarti et al. [24], Ngowi et al. [28], Alexander et al. [19] and Mwidunda et al. [20], were shown to be more effective when community members participated in the design and execution of the interventions. Participatory approaches foster greater community buy‐in, increasing the likelihood of long‐term success. This was confirmed by the present review, where studies that incorporated local participation in designing educational materials or delivery methods showed better outcomes, particularly in terms of knowledge retention and behavioural changes. Conversely, interventions imposed without local input were less successful.

The success of health education in controlling TSCT can also be affected by wider factors, including socio‐economic status, infrastructure and cultural beliefs. Bustos et al. [15] highlighted that many rural communities with limited access to healthcare often do not seek treatment for taeniasis, undermining the effectiveness of health education. Similarly, Ngowi et al. [13] noted that cultural taboos surrounding latrine construction and improper water sanitation were significant barriers to TSCT control in Tanzania. These findings highlight the need to integrate health education with community development initiatives aimed at improving sanitation and access to healthcare. Furthermore, studies suggest that integrating *T. solium* control strategies with other health interventions, such as water sanitation or zoonotic disease control, may enhance their overall effectiveness. The integrated approach could be particularly beneficial in regions with high porcine cysticercosis rates and free‐range pig farming practices.

This review highlights the potential of health education interventions as a key component of TSCT control. For long‐lasting impact, these interventions should be part of a comprehensive control strategy that addresses sanitation, healthcare access and community involvement. Future research, particularly longitudinal studies and randomised controlled trials, is needed to assess the long‐term sustainability of these interventions and their impact on *T. solium* prevalence in endemic areas.

The findings from this review also underscore the importance of incorporating behavioural theories into health education programmes. Montaño and Kasprzyk [45] emphasise that the integration of theories like the Social Cognitive Theory (self‐efficacy) and the Integrated Behavioural Model (IBM) can enhance the effectiveness of interventions by addressing the psychological factors that influence health behaviour. For example, interventions that increase individuals’ self‐efficacy regarding proper handwashing or latrine use, by offering practical solutions or incentives, could have a more lasting impact than programmes focusing solely on awareness.

## 5 Limitations of the Reviewed Studies

Although the studies reviewed offer important insights into the effectiveness of health education interventions for TSCT, they also have several limitations. As previously mentioned, the majority of studies used pre‐ and post‐test designs, which limit the ability to establish causality. Additionally, the variability in study designs (e.g., cluster RCTs and quasi‐experimental designs) makes direct comparisons challenging. Moreover, the lack of standardisation in reporting on key outcomes such as disease prevalence and behaviour change means that conclusions about the effectiveness of health education interventions are somewhat inconclusive. Bieri et al. [44] argue that more rigorously designed studies, with clear outcome measures and longer follow‐up periods, are necessary to draw definitive conclusions about the long‐term impact of health education. Another limitation is that we could not run a meta‐analysis or draw more robust conclusions, for example, due to the lack of quantitative results in some of the studies and indicators for statistical significance in several of the trials. Therefore, we have presented the data from 21 studies in descriptive form only.

## 6 Conclusion and Recommendations

The reviewed studies show that health education interventions delivered through digital platforms, schools, farmer training and community workshops consistently improve knowledge of TSCT transmission and control and often enhance attitudes and practices such as hygiene, pig confinement and reduced open defecation. However, sustained behavioural change and significant reductions in infection prevalence remain inconsistent. Although some interventions achieved notable declines in porcine or HCC, most reported non‐significant changes, reflecting the complexity of breaking transmission cycles. Variations in study design, follow‐up and outcome measures limit comparability and long‐term impact assessment.

Evidence indicates that health education is essential but insufficient alone; integrated, multi‐sectoral approaches are needed. It is therefore recommended: (i) to engage communities in all stages of intervention design; (ii) to apply behavioural theory to understand and sustain change; (iii) to combine health education with sanitation, pig management infrastructure and veterinary inspection; (iv) to extend follow‐up to capture long‐term effects; (v) to standardise KAP and prevalence indicators; (vi) to target multiple stakeholders across the transmission chain; (vii) to conduct more rigorous RCTs with adequate sample sizes and blinded outcome assessment and (viii) to use qualitative and quantitative mixed methods to explore barriers such as cultural practices in pork consumption and pig rearing.

## Author Contributions

**Chacha Nyangi**: conceptualisation, investigation, methodology, validation, formal analysis, data curation, visualisation, writing – original draft, writing – review and editing. **Ernatus Martin Mkupasi**: conceptualisation, investigation, writing – original draft, writing – review and editing, methodology, supervision. **Helena Aminiel Ngowi**: conceptualisation, investigation, funding acquisition, writing – original draft, writing – review and editing, software, formal analysis, methodology, validation, visualisation, project administration, resources, data curation. **Christopher Mahonge**: conceptualisation, methodology, writing – original draft, writing – review and editing, supervision, data curation, validation, visualisation, investigation. **Andrea Sylvia Winkler**: resources, supervision, data curation, software, project administration, writing – review and editing, validation, methodology, writing – original draft, funding acquisition, investigation, conceptualisation, visualisation.

## Funding

This systematic review study was fully funded by the German Federal Ministry of Education and Research under CYSTINET Africa project numbers CYSTINET‐A_1_SUA_81203596 and 01KA1618.

## Disclosure

The funders played no role in the design, conduct or interpretation of the study.

## Conflicts of Interest

The authors declare no conflicts of interest.

## Data Availability

The data that support the findings of this study are openly available in systematic review at https://github.com/Rhobi2008/Systematic‐review.
